# High-throughput analysis of spatio-temporal dynamics in *Dictyostelium*

**DOI:** 10.1186/gb-2007-8-7-r144

**Published:** 2007-07-21

**Authors:** Satoshi Sawai, Xiao-Juan Guan, Adam Kuspa, Edward C Cox

**Affiliations:** 1 Department of Molecular Biology, Princeton University, Princeton, NJ 08544, USA; 2 ERATO Complex Systems Biology Project, JST, Tokyo 153-8902, Japan; 3 Departments of Biochemistry and Molecular and Human Genetics, Baylor College of Medicine, Houston, TX 77030, USA

## Abstract

We demonstrate a time-lapse video approach that allows rapid examination of the spatio-temporal dynamics of *Dictyostelium *cell populations. Quantitative information was gathered by sampling life histories of more than 2,000 mutant clones from a large mutagenesis collection. Approximately 4% of the clonal lines showed a mutant phenotype at one stage. Many of these could be ordered by clustering into functional groups. The dataset allows one to search and retrieve movies on a gene-by-gene and phenotype-by-phenotype basis.

## Abstract 2

A time-lapse based approach is presented that allows a rapid examination of the spatio-temporal dynamics of *Dictyostelium *cell populations, enabling users to search and retrieve movies on a gene-by-gene and phenotype-by-phenotype basis.

## Background

Spatially and temporally evolving collective dynamics act critically to coordinate multicellular development. In general, periodic phenomena are prevalent in transcriptional regulation - for example, in circadian rhythms [1], Msn transcription factor regulation in yeast [2] and the pulsatile response of NF-κB and p53 in tissue culture cells following stimulation [3,4]. Oscillations seem to be a universal mode of regulation for morphogenetic cell movements and gene transcription that requires fine spatial and temporal coordination. Calcium waves are observed during convergent extension in *Xenopus *and are believed to coordinate cell movement [5]. In the case of somitogenesis, where segmentation is periodic, Notch and Wnt signaling is coupled to periodic expression of the Notch components themselves [6,7]. It is expected that the functions of molecular networks will become apparent only when put into the context of such multicellular organization in time and space. Biologically relevant readouts with a temporal and spatial resolution are thus the final layer needed to connect high-throughput genomics data obtained at the molecular and cellular level to higher organizational and functional levels.

A classic experimental paradigm in developmental biology begins with a mutant phenotype and then asks which aspects of development are altered. The goal is to relate structure to function, first at the molecular, then the cellular, and finally the whole-organism level. The current richness of information for a few model organisms is testimony to the success of this approach. With the explosion of genome sequences, it is becoming realistic to rapidly map out relations between genotype and molecular level phenotype using large-scale assays at the level of transcription and translation. Efforts to complement such bottom-up approaches by high-throughput screens based on observational phenotypes at the cellular level have recently been reported in yeast, nematode, and cells in tissue culture [8]. These studies have largely concentrated on the analyses of cell growth, division, and morphology, either through a growth-curve analysis of batch cultures [9,10] or by the analysis of morphology at a single to the few cell level by microscopy [11-15]. However, a comparable approach for a multicellular system based on quantitative real-time dynamic data gathered throughout the entire life cycle remains largely undeveloped.

Here we report on a first attempt in this direction with *Dictyostelium*, where solitary growing cells cooperate upon starvation to form a relatively simple and highly differentiated fruiting body of spore and stalk cells. Pulsatile signaling of the extracellular attractant cAMP, in addition to directing chemotaxis, induces the cAMP signaling components themselves and plays a critical role in determining the size of the aggregation territory [16] as well as coordinating later morphogenesis [17]. We demonstrate that high-throughput profiling of multicellular dynamics detects functional association between developmental genes. We combine collection of movies that covers almost the entire developmental cycle with quantitative and qualitative phenotyping based on temporal data gathered from the movie collection, and parallel genotyping of the characterized clones.

## Results and discussion

### Parallel cell culture and phenotyping

**Figure 1:**
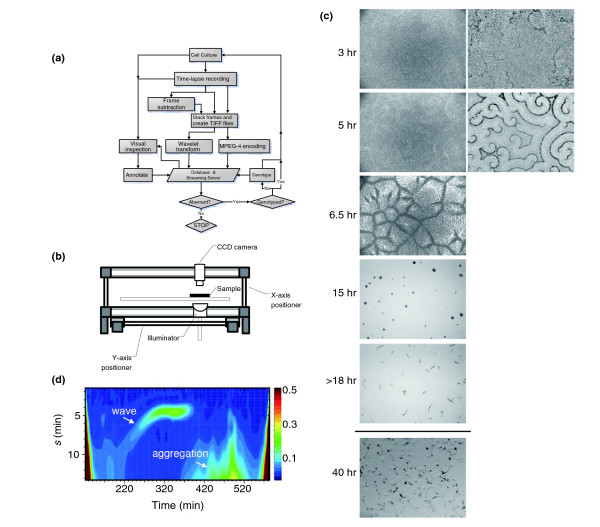
Automated image acquisition and phenotyping of clonal populations. **(a) **Over 2,000 insertional mutant clones were subjected to parallel culture and phenotyping using the flow chart shown here. **(b) **The gantry robotic system. The darkfield optics are positioned below the samples, the digital camera above. **(c) **Snapshots of movies from wild-type AX4 cells at representative stages of development. Images were captured every 40 sec from each well for 10.5 h after plating for a total of 800 frames. Later stages of morphogenesis were then followed for 28.5 h by bright-field illumination. During this period, images were captured at 127-sec intervals, also for a total of 800 frames from each well. The images in the first column were obtained from a 16.8 mm × 12.6 mm area by averaging five frames taken approximately 66 msec apart for noise reduction. Successive averaged frames were then subtracted to obtain the wave images in the second column (3 and 5 h). Bright-field optics were used for the second half of the imaging session to follow slug motion (11 h). After the run was over, the final culminant morphology was checked under a dissecting microscope (48 h). (**d**) The wavelet portrait. For the first 10.5 h, a time course of strength of the signal oscillating at the specified periodicity *s *was obtained from averaged wavelet transformations of pixel intensities as a function of time (see main text for details). Wavelet power spectrum is color coded, and the slow increase in frequency, then abrupt termination, followed by long-period features caused by cell streaming and territory formation, are indicated by arrows.

Cell culture was scaled up to systematically follow the growth and development of as many as a hundred *Dictyostelium *clonal populations at a time (Figure 1a). We designed a robotic system (Figure 1b; see Materials and methods) to capture both early and later events in the morphogenetic cycle. Figure 1c summarizes a typical experiment with our wild-type strain, AX4. Cell-cell signaling mediated by extracellular cAMP was visualized by detecting optical density fluctuations that reflect cell shape change in response to passing cAMP waves [18-20]. By 3 hours after the cells begin to develop, a few fragments of weak optical-density waves have begun to emerge from the background (Figure 1c, 3 hours; see also Additional data file 1 for a movie), a characteristic feature of self-organization in excitable systems [21]. During the next few hours, cells show little directed movement and the cell density is spatially uniform (Figure 1c, 5 hours). The images were enhanced by subtracting consecutive frames (Figure 1c, 3 hours and 5 hours; right panels compared to the left; Additional data file 2). Optical density waves quickly develop spiral cores, which become organizing centers for cell territories by 6.5 hours, when territories of different sizes with aggregating streams of cells are readily apparent (Figure 1c, 6.5 hours; see Additional data file 1). By 15 hours these territories have become rounded masses of cells, the majority of which by 18 hours have reached the motile slug stage, each slug containing from a few thousand to around 10^5 ^cells (Figure 1c, 18 hours; Additional data file 3). By around 40 hours the slugs have migrated and culminated to form fruiting bodies (Figure 1c, 40 hours).

The entire video clip from the first stage of our analysis can be summarized by wavelet analysis, where wave frequency and power spectrum are plotted as a function of time (see Materials and methods) [16]. The wavelet power spectrum (Figure 1d; *z*-axis in pseudocolor) represents the strength of the signal oscillating at the specified periodicity (*s*) (Figure 1d; *y*-axis) as the system develops in time (Figure 1d; *x*-axis). A typical analysis with wild-type cells is illustrated in Figure 1d. At *t *= 150 minutes, long-period (15 min) features have begun to emerge. The wave period evolves slowly and smoothly to *t *= 275 minutes, levels off for 50 minutes, then abruptly switches off as cells migrate to form well defined territories. At approximately *t *= 400 minutes a second long-period feature emerges, corresponding to the cell streaming pattern seen in Figure 1c at 6.5 hours. These results are in good agreement with observations on wild-type cells grown under conventional culture conditions [16], and provide us with a quantitative summary of the first 12 hours of development.

### Phenotype clustering

We have sampled 1,800 insertional mutants, hereafter referred to as the 'unbiased set' from an ongoing large-scale mutagenesis project [22], and 400 or so containing many previously isolated mutants (see Materials and methods). In addition to the quantitative features just described for the early developmental stages, qualitative features such as cell morphology during axenic growth, slug motion/morphology and fruiting body structure (Table 1) were obtained from the movies and observation of the samples by microscopy. From these features, a phenotype matrix *p*_ij _was obtained (see Materials and methods). The matrix is a digital representation of whether or not strains exhibited aberrant behavior at each stage of development.

**Table 1:** Phenotypic characters used in the analysis

Annotated stage	Wild-type features	Examples of mutant features
Growth	Growth, attachment, cell size	Slow growth, no attachment, large cells
Wave	5 min periodicity terminates at 6-7 h after starvation	Slow oscillations, rapid onset, early termination,
Aggregation	Cell streaming with or without late break up	Cell clumping, partial developmental arrest, early break up
Mound	Round mounds giving rise to slugs	Arrest, multiple tips, disintegrating mound
Slug	Migration with a smooth persistent trajectory	Slow migration, arrested migration
Fruiting body	Wild-type culminant structure	Short stalk, long stalk, other aberrant morphology

Phenotype was scored subjectively by comparison of qualitative features of the strain for each stage of development shown above against those of the parental wild-type AX4 strain. For each sample run, these characters were checked by eye from the movies and observation of the samples by microscopy. On the basis of these features, the phenotype matrix *p*_ij _was obtained for further analyses (see Materials and methods).

**Figure 2:**
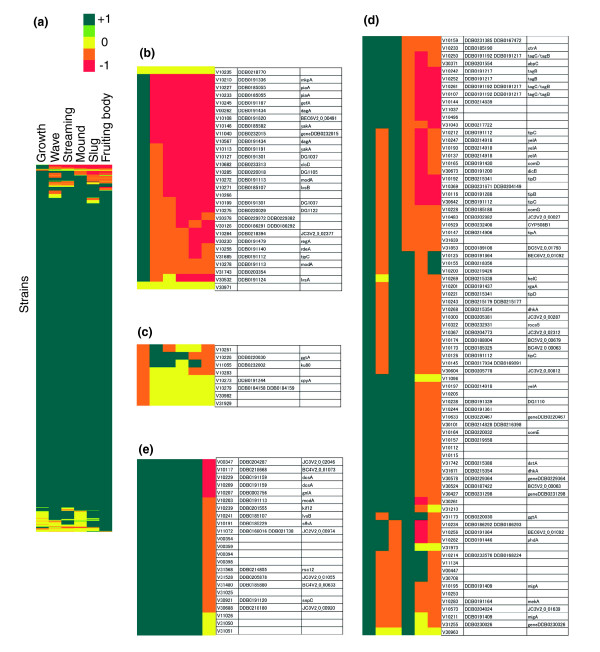
Phenotypic clustering based on the timing of mutant behavior. **(a) **2,257 strains were assigned phenotype vectors according to the stage-specific mutant defect. Color indicates the phenotype index *q*_sj _(see main text for details). A correlation coefficient was used as the phenotype similarity metric. Average linkage clustering was performed on *q*_sj _with zero offset. **(b) **Expanded view of developmentally null and other severely impaired mutant clusters. **(c) **Mid- to late- stage developmental mutant cluster. The table on the right lists the corresponding V-strain IDs in addition to the dictyBase ID and gene name of the disrupted locus. A complete dataset is provided in the form of associated array tree correlations (ATR), complete data table (CDT), gene tree correlations (GTR) and exported raw data files (Additional Data Files 5-8). The movies and other original data can be viewed online by following the hyperlinks provided.

In Figure 2a, the mutants have been categorized on the basis of the phenotype matrix and using a hierarchical clustering method [23]. Our first result is that 83% of the total number of mutant clones (1870 of 2257) cannot be distinguished from wild type (blue-green in Figure 2a), possibly because the insertion is in an intergenic region, or the mutated gene exists redundantly, or it is nonessential for growth and development under the present conditions. The second noticeable feature of these data is the number of strains clustered at the bottom of Figure 2a (139 clones appearing with two or more yellow boxes) and sparsely distributed elsewhere. Many of these exhibited slow vegetative growth and the low cell-density effect associated with it despite multiple attempts to grow them. This phenotype may be largely due to a systematic bias carried over from the parent, as most of them are from the same transformant set. After removing these clones from the dataset, we estimate that 1 to 2% are defective in genes that, while permitting vegetative growth on bacteria, interfere with normal growth in axenic medium. For several mutants in this category, we were able to confirm the observed behavior independently by disrupting the gene by homologous recombination (data not shown). The third feature of this dataset is the remaining strains with developmental phenotypes, representing 4% of the clones in the unbiased mutant set (76 strains out of 1,799) and 32% in the previously characterized mutant set.

Strains that exhibited almost no development, or aberrant behavior throughout all developmental stages, are clustered at the top of Figure 2a (expanded in Figure 2b; *N *= 30). This cluster includes a group of 'developmentally null' mutants in which genes such as *mkpA*, *piaA*, *yakA *and *dagA *are disrupted. Other groups include *DG1105*, *DG1037*, *DG1122 *from an earlier screen (W. Loomis, unpublished work), as well as another group that includes the protein kinase A pathway genes *rdeA *and *regA *(described in detail below). These mutants not only show early developmental defects, they also continue to exhibit aberrant behavior until mound formation, and are either stalled or show further aberrant behavior during slug migration and culmination. The above mutant clusters are followed by a cluster consisted of mutants with similarly severe phenotype plus growth-stage defects (Figure 2c).

Another major mutant cluster contains clones showing defective behavior at the slug and culmination stage, but wild-type behavior during aggregation (Figure 2d). Particularly noticeable are five mutants disrupted in *tagB/C*, mutations in *tipA*, *tipB*, *tipC*, and *tipD*, and multiple occurrence of mutants with insertions in the *yelA *gene and the *dhkA *gene. At the bottom of Figure 2d there are strains that show aberrant behavior in the early stage of development, but nevertheless form mounds, then again exhibit deficient slug and fruiting-body structure. A large number of mutants defective in early signaling are also defective later in development (Figure 2d) even though they appear to stream normally to aggregation centers. This suggests either that the gene products are used at two or more different times during development - for example, cAMP metabolism [24] - or that wave phenotype dictates later aspects of morphogenesis in a way we do not yet fully understand. Finally, some strains exhibited aberrant behavior during the early signaling to aggregation stages, but no striking phenotypes during later stages (see Additional data file 4). These strains may be contrasted with those exhibiting defects only at the slug stage (see Additional data file 4) or the culmination stage (Figure 2e), such as those disrupted in the cellulose synthase gene *dcsA *(Figure 2e).

We noticed that independent clones disrupted in the same gene co-cluster, providing strong validation of our profiling approach. In general, the developmental stages observed for most of the published mutants examined here agree with the literature. Mutants previously characterized as aggregation minus fail to aggregate, and stalk-defective mutants fail to make stalks. A caveat of the present coarse-grained representation is that similarities in the more detailed phenotypes are not reflected in clustering. We should note that detailed phenotypes, such as the break up of aggregation territories seen in chemotaxis-defective mutants of *erkA *[25], *mekA *[26] and *phdA*, [27] and long stalks in *dhkA *[28] also agree well with known mutant phenotypes.

However, not all of the phenotypes were consistent with the literature. This includes V31742 from the new unbiased mutant set carrying an insertion in *dstA*, a gene encoding the STATa transcription factor, which under our assay conditions was defective only from the slug stage on, whereas a delay earlier in development has been reported [29]. There were also some that exhibited phenotypes undocumented in the literature. For the two most conspicuous clones (disrupted in *splA *and *lvsB*), we showed that the phenotype could not be recapitulated by an independent knockout. In these cases, a secondary mutation introduced by the REMI vector is the likely cause of the observed defects. While it is possible that some of the differences between independent isolates are due to subtle differences in cell density and the growth condition at the outset of each experiment, we note that phenotyping was repeated two or more times, and thus it is likely that the clustering reflects either differences traceable back to mutant gene structure or the highly plastic nature of the mutant phenotype (for example, *tipC*, *modA*, *yelA*).

### Early wave features

**Figure 3:**
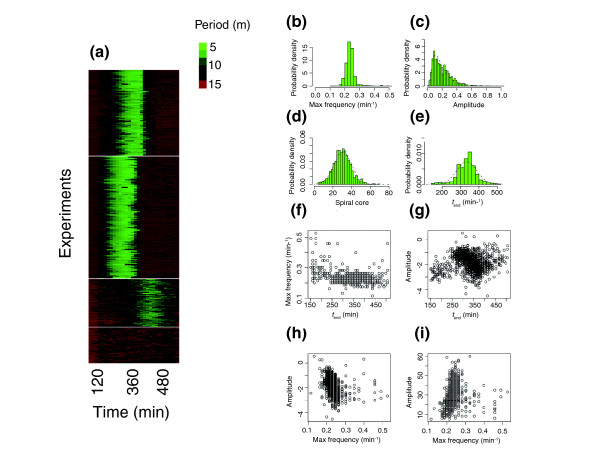
Early cell-cell signaling. **(a) **The wavelet transform was further reduced to a one-dimensional representation by tracing the peak of the averaged wavelet power spectrum as a function of time *t*. The traced data were then subjected to K-mean clustering. The bottom cluster comes from experimental runs where the normal 5-min optical-density oscillations were not detected. Other clusters are wild type with respect to signaling periodicity but are grouped according to the difference in wave onset. The second cluster from the bottom shows large deviations in the timing and consists mainly of samples with low cell density. **(b) **The frequency of the optical-density oscillations before termination is narrowly distributed and highly reproducible. **(c) **The wavelet power spectrum, on the other hand, follows a log-normal distribution. **(d) **The number of spiral cores in an area of 2.1 cm^2 ^and **(e) **the time of cessation of the periodic signaling follow a Gaussian distribution (shown as a dashed curve). **(f-i) **Scatter plots indicate relations between these measures that reflect properties of the self-organizing pattern formation from random initial conditions (see main text for details). Correlation coefficients are (f) -0.20, (g) 0.05, (h) -0.37 and (i) 0.15 respectively. Original data are provided as Additional data files 9-13.

Several wavelet parameters serve to characterize the wild-type phenotype of early cAMP signaling. The peak of the averaged wavelet power spectrum was traced, and the time of the cessation of signaling *t*_end _was determined. The resulting one-dimensional data can be clustered, yielding a group of samples that failed to exhibit normal oscillation patterns (Figure 3a, and see the next section). We have done this by first placing sample runs into four groups using K-mean clustering of the wavelet transform (see Figure 3a), then removing possible pleiotropic effects during the growth phase by cross-verification with the phenotype cluster. A similar analysis using hierarchical clustering yields a continuous profile without apparent structure or organization. The first two clusters in Figure 3a contain samples with slight differences in the onset that is within that observed in the wild type. The third cluster in Figure 3a, with delayed wave-onset time, contains mostly low-density samples, whereas samples in the last cluster failed to establish waves. There is a faint secondary peak above the first peak in the wavelet portrait that signifies a deviation from the symmetric sinusoidal form of oscillation. Although these secondary peaks may be important to characterize mutants with altered forms of oscillation, such as *stmF *[30], we have confined our analysis here to the main frequency.

On the basis of the samples that fell into the top three clusters in Figure 3a, we sought to obtain the distribution of the cAMP wave phenotype in order to gain insights into the underlying self-organizing mechanism. At *t *= *t*_end_, the main frequency 1/*s *= 1/s* and the peak wavelet power spectrum was extracted. Figure 3b records the distribution of maximum frequency 1/s* of the optical density oscillations. The maximum frequency is narrowly distributed, with an average of 0.24/min (standard deviation (SD) ± 0.03). This is equivalent to cells reaching approximately a 4.2-min period oscillation, in agreement with previous studies [18,31,32]. Compared with the tight distribution of signal frequencies, the wavelet power spectrum follows a log-normal distribution, with mean 0.197 (SD ± 0.132) (Figure 3c). Cessation of oscillations *t*_end _is well fitted by a Gaussian distribution (Figure 3e). A tight frequency distribution and a broad (log-normal) amplitude distribution have also been reported recently in the p53 system [33] and may be a widespread feature of nonlinear oscillations in cells.

The number of spiral wave cores, which is a good measure of the number of cell territories that will later form, also follows a Gaussian distribution (Figure 3d). This distribution is most plausibly explained by the fact that core formation is intrinsically stochastic in nature [16,34]. It is also likely that the observed distribution depends on sample to sample variability in cell density which may correlate with the oscillation frequency (see below), although the number of aggregation centers is known to be relatively insensitive to cell density above 400 cells/mm^2 ^[35], and our experiments were carried out at around 7,000 cells/mm^2^. To exclude such complications, the data in Figure 3b-i were obtained from selected samples exhibiting spiral wave propagation where the growing cells had reached confluence and showed no growth defects (*N *= 1,639; top three clusters in Figure 3a). We noticed that the number of aggregates exceeds the number of spiral cores because streams tend to break up just before aggregation completes. The extent of late stream break up was highly variable from sample to sample, even for the same strain, and therefore this phenotype was not considered as a robust trait for further annotation.

Spiral wave formation is a complex phenomenon that depends on the developmental trajectories of the cells; that is, how the mode of signaling [36], sensitivity to the signal [16] and kinetics of signaling [32] develop in time. We investigated this aspect by displaying the related data as scatter plots (Figure 3f-i). We note the following. First, when the system develops quickly, there is a weak tendency for the oscillation frequency to be smaller (Figure 3f). Second, there appears to be a weak positive correlation between the amplitude and *t*_end _(Figure 3g) and a negative correlation between the amplitude and the frequency at *t*_end _(Figure 3h). Heterogeneity in the signaling response has been reported at the single cell level [37,38]. Because our analysis is based on data from groups of cells, wavelet amplitude mainly reflects the coherence among the cells of the periodic cytoskeletal rearrangement upon cAMP stimulation. The data, therefore, suggest that the cells are participating in periodic signaling more heterogeneously when the system takes a shorter time to reach the streaming stage, and/or when it reaches a high-frequency oscillation state. We see that in high-frequency samples, more spiral cores are observed (Figure 3i). From the slope, there is roughly a fivefold increase in maximal number of spiral cores as the frequency increases from 0.17/min to 0.25/min. This is difficult to explain simply by scaling of the territory pattern with wavelength alone, because one can only expect an increase of approximately 1.5-fold. Rather, the data suggest a causal relationship between the formation of spiral cores and heterogeneity in cell excitability.

### Pulsing and slow-oscillator mutants

**Figure 4:**
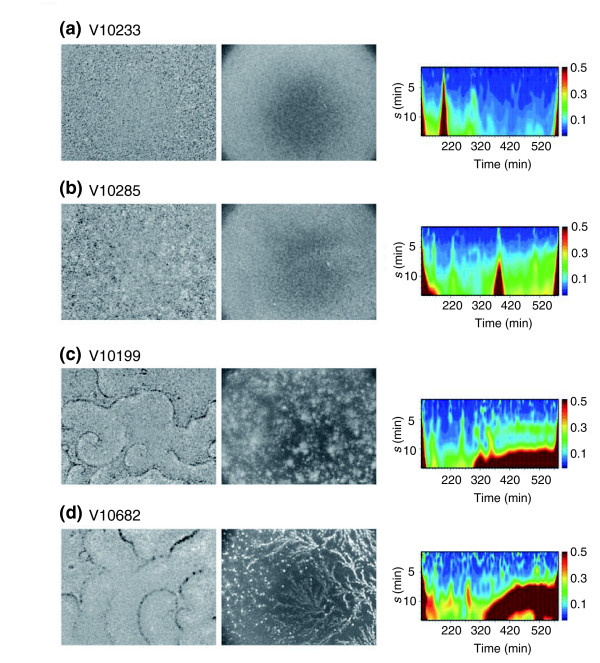
Representative samples with defects in early development. The severity of the signaling phenotype ranges from the absence of optical-density waves to delayed slow oscillations. Frame-subtracted images at *t *= 6-8 h are shown on the left and the original images at *t *~10 h are shown in the center. Wavelet portraits are on the right. **(a) **V10233 (*pia*A) shows no sign of periodic signaling. **(b) **V10285 (*DG1105*) shows local pulsatile activity, whereas **(c) **V10199 (*DG1037*) and **(d) **V10682 (*clc*D) are slow oscillators with incomplete aggregation or delayed aggregation, respectively. Data shown are from mutant clones recreated by homologous recombination.

As described above, early-stage mutants that failed to exhibit the typical developmental time course in optical-density oscillations can be systematically picked up by the clustering of the wavelet transform (Figure 3a, the bottom cluster). The mutants detected in this way display a range of severity in signaling defects. For example, V10233 (Figure 4a) is disrupted in the *piaA *gene, which encodes a TOR (target of rapamycin) complex protein that is required for the cAMP pulse-induced activation of adenylyl cyclase [39]. Neither optical-density waves nor signs of aggregation are visible, as expected from the known null phenotype of *piaA *mutants. V10285 (*DG1105*; dictyBase ID: DDB0220018) shows local pulsatile waves, and development at this stage is prolonged (Figure 4b). V10199 (*DG1037*; dictyBase ID: DDB0191301) shows slow oscillations of extended duration (Figure 4c), and development appears to be arrested during early aggregation. Owing to the long period of the optical-density oscillations, the wavelength of the spirals is extended, and therefore only a few spiral wave territories appear. Finally, V10682 is able to develop after growth and starvation on bacterial plates, but on non-nutrient agar, development is delayed from early aggregation on (Figure 4d). Optical-density wave onset is late, and wave periodicity remains long and never reaches the characteristic 5-min oscillation. The gene disrupted in this strain (dictyBase ID: DDB0218077) encodes a protein homologous to the conserved *clc6/7 *type chloride channel family protein [40].

### PKA pathway mutants and optical-density waves

**Figure 5:**
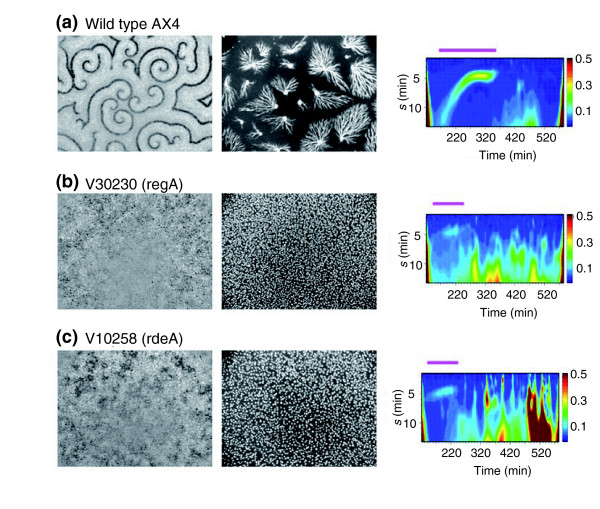
The screen identifies mutants with accelerated development. Frame-subtracted images at *t *= 2-4 h (left) and the raw images at *t *= 5-8 h (center). Wavelet portraits are shown on the right. **(a) **Wild-type AX4; **(b) **V30230 (*reg*A); **(c) **V10258 (*rde*A). The signaling period is emphasized by the magenta bar above each portrait.

In contrast to the mutants described above, all of which are strongly defective in early signaling, two strains (V10258 and V30230) that exhibit notably altered wave and aggregation phenotype (Figure 5b, c) are found together in the clustered array (Figure 2b). In these mutants, waves propagate for very short distances before annihilating when they crash into each other. Compared with wild-type behavior (Figure 5a), periodic signaling begins early in both strains, and the signaling duration is abbreviated to 1 hour (Figure 5, magenta bar in right panels). Cells aggregate precociously, forming small mounds with very little evidence of streaming toward a spiral center. Furthermore, the aggregation process is completed in 3 hours. These features are clearly seen in the wavelet analysis (Figure 5, right panels). We note the striking similarity of the wavelet portrait for these two strains.

Strain V30230 and V10258 carry an insertion in the *regA *and *rdeA *genes, respectively. The *regA *gene encodes an intracellular cAMP phosphodiesterase with a response regulator domain at the amino terminus [41,42], and the *rdeA *gene encodes the only known histidine phosphotransfer domain protein in *Dictyostelium discoideum*. A biochemical study has shown directly that a receiver domain of RdeA relays phosphate groups to the amino-terminal response regulator domain of RegA and that phosphodiesterase activity of RegA is stimulated by phosphorylation of the amino-terminal receiver domain [42]. We have recently shown that PKA pathway mutants show similar crowded-wave phenotypes due to the emergence of abnormally large numbers of spiral cores, and thus this independent isolation of insertions in *rdeA *and *regA *is an important confirmation of a recent model of pattern formation that incorporates coupling of external cAMP oscillations to internal cAMP levels [16]. Other genotyped mutants related to this pathway were those with insertions in *dhkA, dhkC, dhkJ *and *acrA*. Mutants in *dhkC *(V10588) show early slow waves reminiscent of other previously studied PKA pathway mutants *pkaR*^- ^[16] or *dhkK *(D1125N) [43] (data not shown). In contrast, *dhkA *and *acrA *show mutant phenotypes only at later stages consistent with their specific roles during slug to culmination stage. A mutant in *dhkJ *was found in the wild-type cluster.

### Slug mutants

The slug is a multicellular structure consisting of anterior prestalk cells and posterior prespore cells that migrates towards favorable environments for culmination. Studies suggest that propagating waves of cAMP not only direct cell aggregation during the early stage of development, but may also coordinate cell migration in the slug stage [17,44]. Slug migration velocity is typically of the order of several hundred micrometers per minute; therefore its characterization is difficult without time-lapse imaging.

**Figure 6:**
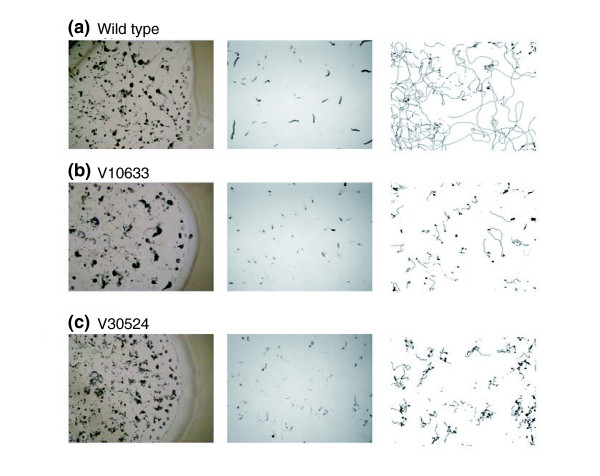
The screen uncovers mutants with aberrant slug motion. The multicellular slug phenotype is often difficult to see in cells feeding on bacterial lawns (left-hand panel) because development is asynchronous and the slug stage is transient. The middle panels are snapshots from our automated imaging system taken at around 24 h. Slug trajectories over a 28.5-h period were obtained by first binary thresholding the movies and then tracking the center of mass by multiple particle tracking using ImageJ (right-hand panel). Data shown are from mutant clones recreated by homologous recombination.

Our dynamical profiling approach reveals mutants with coordination defects. A mutant V10633 of a putative GATA activator (dictyBase ID: DDB0220467) forms chubby slugs that are mostly developmentally arrested at this stage (Figure 6b, right panel). Migration is almost absent, as is evident from the slug trajectories (Figure 6b right panel). Some slugs do culminate to form fruiting bodies with small spore heads. The video records allow one to discriminate mutants with such behavior from those that proceed to the slug stage but show deficient migration. In V30524 (Figure 6c), the slugs move with less path persistence compared to wild type (Figure 6a). V30524 carries an insertion in an open reading frame (dictyBase ID: DDB0187422) that encodes an arginine-N-methyltransferase, a conserved PRMT5 family protein involved in post-translational modification of proteins involved in RNA processing, DNA repair, and transcriptional regulation [45].

Note that we did not base our phenotypic scoring on the ability of slugs to sense light or thermal gradients, and therefore we have probably missed genes implicated in these processes for example, *gefL *[46] (Additional data file 5). Another phototaxis mutant that was nevertheless scored (Figure 2d, *abpC*) may be more severely impaired in morphogenesis because of other defects [47].

## Conclusion

We have shown that parallel phenotyping in a screen based on macroscopic multicellular dynamical features of over 2,000 clonal *Dictyostelium *populations is possible in a relatively short time by combining parallel cell culture, automated high-throughput time-lapse imaging, and quantitative and qualitative phenotyping of multicellular behavior. The time-lapse movies contain a wealth of information that reflects the ability of individual cells to attach to the substratum, signal to one another, perform directional movement towards an attractant, form a multicellular body, migrate as a whole, and differentiate to construct the final culminant. In this study, we have shown how such a readout can be obtained for a simple multicellular organism, annotated, and stored in a form of streaming video that can easily be linked to a genome database [48].

The current study achieved a comparative assay of mutant phenotype under uniform environmental conditions. We showed that mutants disrupted either in the same gene, genes in a common signal transduction pathway, or genes known to cause a similar morphological defect, such as mutants in *tip *gene*s *[49], can be clustered solely on the basis of a Boolean matrix of the affected developmental stage without any reference to the specific defects observed. The number of major mutant cluster categories was on the order of the number of developmental stages, *N*_i_. Assuming random insertion in the mutagenesis, the expected number of developmental genes in each cluster (*N*_g_) is approximately *N*_g _= (*G *× *P*)/(*N *× *r*) where *G *is the number of genes in the genome, *P *is the mutant frequency and *r *is the frequency of the coding regions in the genome. We found *P *= 0.04, which is larger than an estimate of 0.3-1% of the clones exhibiting visible developmental aberrations [50], suggesting increased sensitivity of mutant detection by our current scheme. Substituting the predicted number of genes in the genome [51] (*G *≅ 1.25 × 10^4^; *r *= 0.7) we estimate a total of 720 genes which when disrupted should exhibit a mutant phenotype during development under our assay, and that a major mutant cluster should on average comprise around 120 genes. This is in line with an estimate of 100-150 genes essential for early development [52]. Our total estimate of developmental genes in *Dictyostelium *is double the earlier estimate of 300 genes [52], and about half the number of genes reported to affect zebrafish morphogenesis [53].

Multicellularity is achieved through the coordinated action of cellular processes such as cell growth and death, cell-cell signaling, cell movements, and cell adhesion, which leads to differentiation of cell types and morphogenesis of a multicellular structure. Although the transitions from unicellular eukaryotes to multicellular ones seem to have occurred independently many times during the course of evolution [54,55], what we know about the requirement for such transitions is very limited [56,57]. How many genes are necessary? What form of networks of genes and proteins are required? When combined with other systematic phenotype analyses [58,59] and sequencing of related social amoeba species now under way, a complete time-lapse movie set and functional grouping of knockout mutants of every gene in the *Dictyostelium discoideum *genome would have a major impact on our understanding of life-cycle evolution.

## Materials and methods

### Cell culture

Clones of random insertional mutants generated by restriction-enzyme-mediated insertion (REMI) [60] and wild-type *D. discoideum *cells were grown on fresh lawns of *Klebsiella aerogenes *on SM agar for 3 to 4 days. The cells were picked from a feeding-front of a plaque into 2 ml growth medium (PS medium 1 l; 10 g Special Peptone (Oxoid, Basingstoke, UK), 7 g Yeast Extract (Oxoid), 15 g d-glucose, 0.12 g Na_2_HPO_4_·7H_2_O, 1.4 g KH_2_PO_4_, 40 μg vitamin B12, 80 μg folic acid) supplemented with 1 × Antibiotic-Antimycotic (Gibco; Invitrogen, Carlsbad, CA). Typically, 30 clones were cultured in parallel using five six-well plates (Costar 3506; Corning, Lowell, MA). After incubation at 22°C for one day, bacteria and other debris were removed by gentle shaking followed by aspiration of the medium. Fresh PS medium was then added and the cell density was readjusted if necessary. The cells were allowed to attach to the bottom of the plate and incubated at 22°C for another 24 h. Cell density in the initial inoculation was typically 2 × 10^6 ^cells/well. Under these conditions, wild-type AX4 cells attach robustly to the plate surface and appear non-polarized. They grow and divide about three times at a doubling time of approximately 12 h before reaching confluency at 7 × 10^6 ^cells/well.

Growth medium was then removed and the cells were resuspended in 1 ml DB (10 mM KH_2_PO_4_/Na_2_HPO_4_, 2 mM MgSO_4_, 0.2 mM CaCl_2_; pH 6.5) and transferred to a 1% agar (Gibco Bactoagar) surface prepared in six-well plates where they were allowed to settle for 15 min to form a monolayer. Supernatant was removed and the plates were allowed to dry for 15 min in a sterile hood.

### Mutant clones

The life cycle of 2,257 mutagenized clones was analyzed. Clones were of two major types. To test the generality of our approach, we analyzed a collection of mutants generated by restriction-enzyme-mediated insertional (REMI) mutagenesis, many of which have been published. These strains came from the Loomis and Shaulsky laboratories. They are numbered V00262 to V10300. To test our methods to discover new mutants with developmental phenotypes by unbiased random REMI mutagenesis, we analyzed a subset of an extensive collection developed at Baylor. This is the V10301-V11139 and V30000-V31999 series. Whenever the phenotype deviated from wild type, the time-lapse experiment was repeated, with the result that 882 clones were examined more than once. Of these, 357 were repeated two or more times. REMI mutagenesis provides a convenient and relatively unbiased way to conduct genome-wide forward genetic screens, allowing the investigator to rapidly identify the insertion site by plasmid rescue and inverse PCR. The insertion sites for the entire set were determined at Baylor University [22]. Those with suspected aberrant phenotypes were resequenced at Princeton University on a strain-by-strain basis (see below).

### Plasmid rescue, inverse PCR and homologous recombination

Genomic DNA was prepared by a salting-out method [61] from cells shaken overnight in phosphate buffer (20 mM KH_2_PO_4_/Na_2_HPO_4 _pH 6.5). Approximately 1 μg of DNA was cut with the six-cutter restriction enzymes *EcoR*I, *Cla*I, *Bgl*II or *Spe*I (New England Biolabs, Ipswich, MA). Digested DNA was electrophoresed in 1% TAE agarose gels and used for Southern blot analysis. Plasmid pBSRΔ Bgl was cut with *BamH*I and *Hind*III and the resulting 1.4-kb fragment containing the Blasticidin resistance cassette [62] was P^32^-labeled and used as a probe. Digests that yielded a specific band of 6 to 12 kbp were chosen for plasmid rescue. DNA (6 μg) was cut and then circularized using T4 DNA ligase (New England Biolabs). The ligation reaction was purified and used to transform electro-competent SURE cells (Stratagene, La Jolla, CA). Clones were selected on LB ampicillin plates and three clones were typically picked for plasmid DNA preparation and sequencing. Sequencing reactions were performed from both ends of the inserted vector pBSR1 [63] using T7 and SP6 primers.

For inverse PCR, approximately 100 ng of DNA was first digested with RsaI, which recognizes sites close to both ends of the inserted vector pBSR1. The digest was heat inactivated, purified using silica columns (PCR purification kit; Qiagen, Valencia, CA) and circularized with T4 DNA ligase. Using the circularized DNA as a template, two PCR reactions were performed to amplify flanking DNA from the ends of the inserted vector. The primers for the T7 end were (T7) 5'-TAATACGACTCACTATAGGG-3' and (InvT7R2) 5'-CTGCACTACCAATCGCAATGG-3'. For the SP6 side, they were (InvSp6L) 5'- GCCGCGTTCTAACGACAATA-3' and (InvSp6R) 5'-TCATACACATACGATTTAGGTGACA-3'. Positive PCR reactions were purified using silica columns (PCR purification kit; Qiagen) and sequenced using T7 or SP6 primers. For some PCR samples, sequencing reactions were performed after cloning the PCR product into TOPO pCR2.1 vector (Invitrogen). The sequence was parsed and BLAST-searched against the *Dictyostelium *chromosome sequence using EMBOSS [64] with a script written in Perl [65] and then manually inspected to identify insertion positions.

We were able to identify the insertion sites of the vector for approximately 80% of the 300 clones that were examined more than once. For the other 20%, Southern analysis revealed that there were no appropriate six-cutters available for plasmid excision, and/or where inverse PCR failed. Of the 344 different clones genotyped in Princeton, the two ends of the inserted vector were found at separate loci in 42 cases. Such anomalous insertion events have also been reported following REMI mutagenesis in *Saccharomyces cerevisae *[66]. For those mutants described in detail, gene disruption was repeated by homologous recombination using the isolated plasmid obtained by the methods described above. Wild-type AX4 was electroporated with the linearized plasmid following a standard protocol [67]. Positive clones were selected in PS medium supplemented with 10 μg/ml Blasticidin S (MP Biomedicals, Solon, OH) and recombination was verified by PCR.

### Time-lapse imaging and database constuction

An imaging robot was constructed using industrial automation assemblies. It is a gantry system, with two *x*-*y *instrument platforms ganged together, one positioned above the sample holding area, the other below, each driven by digital servo drives (Gemini GV; Parker Automation, Cleveland, OH) (Figure 1b). The drives are operated through a programmable two-axis servo controller (6K2; Parker Automation). The servo tuning and axis-control programs were written using Motion Planner software (Parker Automation). The upper gantry platform houses a 1/3-inch format CCD camera (LCL-903HS; Watec, Orangeburg, NY) with a macro lens. The dark-field illumination optics consisting of a fiber-optics light guide and lenses, is mounted on the lower platform. Although this is a belt-driven system, feedback loops in the controllers allowed positioning over the 2 m × 2 m sample platform with a reproducibility of around 100 μm rms (root mean square). Time interval fluctuation measured at a single well for both the first and second time intervals was typically 0.1 sec (standard deviation). The robot was housed in a light-tight room at a constant temperature of 22°C. Six-well plates were placed on a stage that can hold up to 100 accurately aligned in the *x-y *plane. Images from a 16.8 mm × 12.6 mm area from each well were captured and transferred to a computer, where they were digitized and stored in 640 by 480 pixel 8-bit grayscale TIFF format using a frame grabbing board (LG-3; Scion Corporation, Frederick, MD). Image files were written to a high capacity hard-disk system (Xserve RAID; Apple Computer).

Image acquisition, frame stacking and frame subtraction were accomplished using Java-based plug-in applications written for ImageJ [68]. These files were encoded in MPEG-4 format using ImageJ and Quicktime Pro (Apple Computer) for easy viewing over the Internet using a streaming server. Subtracted movie files were encoded at 12 frames/sec. The first and second half of the original movies were encoded at 48 and 36 frames/sec, respectively. Movie files, wavelet data and annotation data were stored on a MySQL server. Data acquisition, data management and statistical analyses using the MySQL database were performed with web-based queries written in PHP and the R statistical package [69]. Raw data can be found on our website [70].

### Wavelet transform and phenotype clustering

Wavelet analysis was performed as described [16] with some modification. Briefly, from the original TIFF movie files, time-series *ρ *(*x*, *y*; *t*) of average pixel intensity from 3 × 3 pixel areas at coordinate (*x*, *y*) were sampled from a mesh of 20 pixel intervals (*M *= 2,048 sites). From the time-series, normalized wavelet power spectra averaged over space were obtained by

$$\bar{|W(s,t){|}^{2}}=\frac{1}{M}\sum_{x,y}\frac{1}{\sigma_{\text{xy}}^{\text{ 2}}}{\left|\sum_{n'=0}^{N-1}\rho (x,y;t)\psi \{(n'-n)\Delta t/s\}\right|}^{2}$$

where Δ*t *is the time interval of the time series *ρ*(*x*, *y*; *t*) with variance σ_xy_^2^, and *ψ *is the Morlet wavelet:

*ψ*(*η*) = *π*^-1/4 ^exp[i*ω*_0_*η*-*η*^2 ^/2]

where *ω*_0 _= 6. These procedures were automated and integrated with image acquisition. Feature extraction from the wavelet analysis was performed using a script written in Perl that traces the peak of the wavelet power spectrum as a function of time. A running average with a time interval of 6.7 min was used to remove short time-scale fluctuations. The resulting trace data were clustered using a K-means algorithm with the Euclidean distance as a similarity metric.

For each developmental stage - growth, early wave, aggregation, mound, slug and fruiting body - deviation from reproducibly robust wild-type behavior at each stage was noted (Table 1). This information was ranked into four different categories *p*_*ij *_= -1, -1/2, 0 or 1, where *i *∈ [1,6] stands for ordered developmental stage (for example, *i *= 1 is the growth stage) and *j *represents the sample number. Category *p*_*ij *_= 1 is thus the value for a given wild-type phenotype, and *p*_*ij *_< 1 signifies the severity of the mutant phenotype. *p*_*ij *_= -1 corresponds to a null phenotype, meaning that a developmental stage-specific behavior and morphology was completely absent, either because of developmental arrest at that particular stage, or at a preceding stage. *p*_*ij *_= -1/2 is assigned when a clear deviation from wild-type behavior could be identified (for example, slow oscillations, short stalk, and so on). A phenotypic score of *p*_*ij *_= 0 was assigned when the phenotype could not be distinguished from phenotypic fluctuations exhibited from experiment to experiment with wild-type cells. Many clones (V10546-V10646, V10676-V10696, V30001-V30896, V31301-V31596) systematically showed late slug behavior characterized by loss of cells from the slug posterior and early culmination. These were assigned *p*_*ij *_= 0 and treated as wild type for clustering purpose. Multiple sample runs were averaged by taking the maximum

$$q_{sj}=\overset{Ns}{\underset{i=1}{\max}}p_{ij}$$

for strain *s *with *N*_*s *_repeated runs. Although this filtering approach loses some information relative to simple mean averaging, it supplies a more rigorous justification for the claim that a given strain is defective in some aspect of development. Hierarchical clustering was performed by Cluster 3.0 [71] using Pearson correlation as a similarity metric. The mean of all pairwise distances were used during clustering. The resulting trees were visualized by Java TreeView [72].

## Additional data files

Additional data is available online with this paper. Additional data file 1 is a time-lapse movie in MPEG-4 format of wild-type AX4 taken during the first 10 hours of development. Additional data file 2 is a video in MPEG-4 format consisting of frame-subtracted images of the data shown in Additional data file 1. Additional data file 3 is a time-lapse movie in MPEG-4 format of the later stages of development of the same sample as shown in Additional data file 1. Additional data file 4 is a figure giving a blow-up view of other mutant clusters found in Figure 2a. Additional data files 5, 6, 7 need to be in a folder before opening the CDT file (Additional data file 6) with Treeview. For a link to the movies, paste into Java Treeview (under Settings→Presets→Gene Url Presets) the URL . Additional data file 5 is a GTR file for Figure 2a. Additional data file 6 is a CDT file for Figure 2a. Additional data file 7 is a JTV file for Figure 2a. Additional data file 8 is the original raw data plus hyperlink to the online movies for Figure 2a. The value of phenotype index is doubled for easier viewing in Java Treeview. Additional data files 9, 10, 11 need to be in a folder before opening the CDT file with Treeview. Additional data file 9 is a KGG file for Figure 3a. Additional data file 10 is a CDT file for Figure 3a. Additional data file 11 is a JTV file for Figure 3a. Additional data file 12 is the raw data for Figure 3a. Additional data file 13 is a database file for Figure 3b-i.

## Supplementary Material

Additional data file 1 A time-lapse movie of wild-type AX4 taken during the first 10 hours of development. Click here for file

Additional data file 2 A video consisting of frame-subtracted images of the data shown in Additional data file 1. Click here for file

Additional data file 3 A time-lapse movie of the later stages of development of the same sample as shown in Additional data file 1. Click here for file

Additional data file 4 A figure giving a blow-up view of other mutant clusters found in Figure 2a. Click here for file

Additional data file 5 A GTR file for Figure 2a. Click here for file

Additional data file 6 A CDT file for Figure 2a. Click here for file

Additional data file 7 A JTV file for Figure 2a. Click here for file

Additional data file 8 The original raw data plus hyperlink to the online movies for Figure 2a. Click here for file

Additional data file 9 A KGG file for Figure 3a Click here for file

Additional data file 10 A CDT file for Figure 3a Click here for file

Additional data file 11 A JTV file for Figure 3a Click here for file

Additional data file 12 Raw data for Figure 3a. Click here for file

Additional data file 13 Database file for Figure 3b-i. Click here for file
