# Supplementary material for: High-throughput analysis of spatio-temporal dynamics in Dictyostelium
Source: Genome Biol. 2007 Jul 21;8(7):R144. doi: 10.1186/gb-2007-8-7-r144 (PMC2323234; doi:10.1186/gb-2007-8-7-r144)
Supplement: Additional data file 4 — A figure giving a blow-up view of other mutant clusters found in Figure 2a. [file gb-2007-8-7-r144-S4.pdf]

Supplementary Figure

S. Sawai et al (2007) “High-Throughput Analysis of Spatio-temporal Dynamics in Dictyostelium”

a

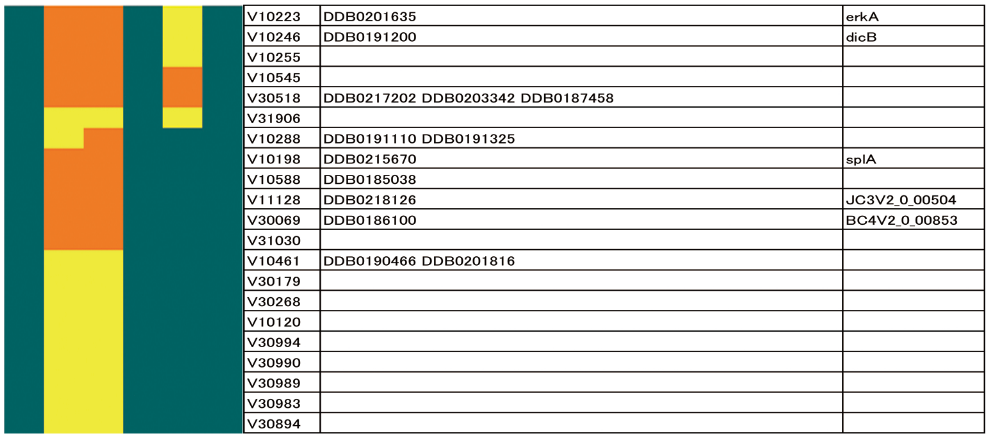

V10223	DDB0201635	erkA
V10246	DDB0191200	dicB
V10255		
V10545		
V30518	DDB0217202 DDB0203342 DDB0187458	
V31906		
V10288	DDB0191110 DDB0191325	
V10198	DDB0215670	splA
V10588	DDB0185038	
V11128	DDB0218126	JC3V2_0_00504
V30069	DDB0186100	BC4V2_0_00853
V31030		
V10461	DDB0190466 DDB0201816	
V30179		
V30268		
V10120		
V30994		
V30990		
V30989		
V30983		
V30894		

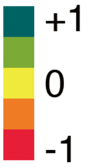

b

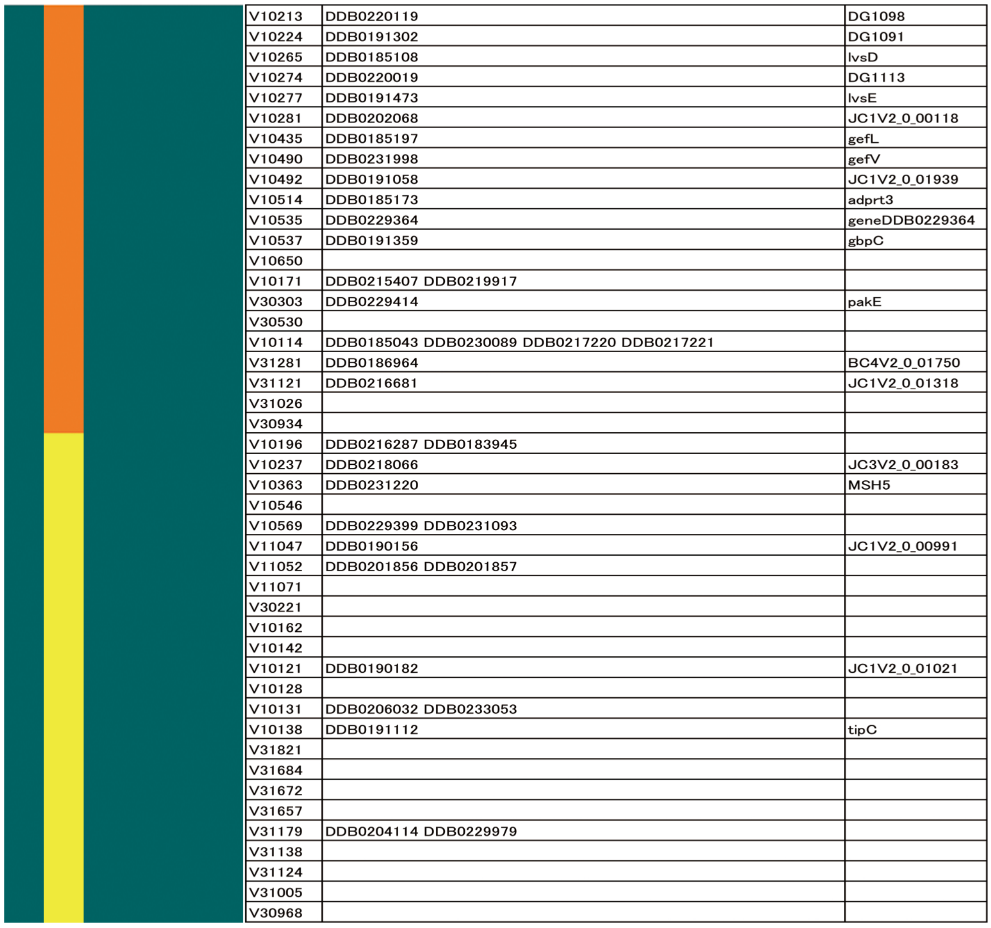

V10213	DDB0220119	DG1098
V10224	DDB0191302	DG1091
V10265	DDB0185108	lvsD
V10274	DDB0220019	DG1113
V10277	DDB0191473	lvsE
V10281	DDB0202068	JC1V2_0_00118
V10435	DDB0185197	gefL
V10490	DDB0231998	gefV
V10492	DDB0191058	JC1V2_0_01939
V10514	DDB0185173	adprt3
V10535	DDB0229364	geneDDB0229364
V10537	DDB0191359	gbpC
V10650		
V10171	DDB0215407 DDB0219917	
V30303	DDB0229414	pakE
V30530		
V10114	DDB0185043 DDB0230089 DDB0217220 DDB0217221	
V31281	DDB0186964	BC4V2_0_01750
V31121	DDB0216681	JC1V2_0_01318
V31026		
V30934		
V10196	DDB0216287 DDB0183945	
V10237	DDB0218066	JC3V2_0_00183
V10363	DDB0231220	MSH5
V10546		
V10569	DDB0229399 DDB0231093	
V11047	DDB0190156	JC1V2_0_00991
V11052	DDB0201856 DDB0201857	
V11071		
V30221		
V10162		
V10142		
V10121	DDB0190182	JC1V2_0_01021
V10128		
V10131	DDB0206032 DDB0233053	
V10138	DDB0191112	tipC
V31821		
V31684		
V31672		
V31657		
V31179	DDB0204114 DDB0229979	
V31138		
V31124		
V31005		
V30968		

c

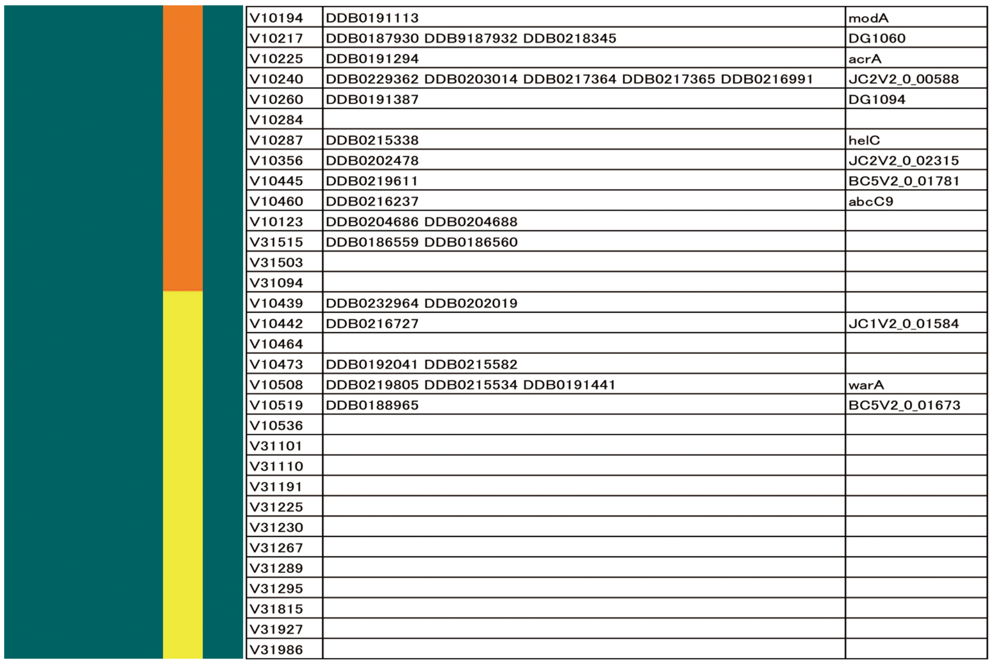

V10194	DDB0191113	modA
V10217	DDB0187930 DDB9187932 DDB0218345	DG1060
V10225	DDB0191294	acrA
V10240	DDB0229362 DDB0203014 DDB0217364 DDB0217365 DDB0216991	JC2V2_0_00588
V10260	DDB0191387	DG1094
V10284		
V10287	DDB0215338	helC
V10356	DDB0202478	JC2V2_0_02315
V10445	DDB0219611	BC5V2_0_01781
V10460	DDB0216237	abcC9
V10123	DDB0204686 DDB0204688	
V31515	DDB0186559 DDB0186560	
V31503		
V31094		
V10439	DDB0232964 DDB0202019	
V10442	DDB0216727	JC1V2_0_01584
V10464		
V10473	DDB0192041 DDB0215582	
V10508	DDB0219805 DDB0215534 DDB0191441	warA
V10519	DDB0188965	BC5V2_0_01673
V10536		
V31101		
V31110		
V31191		
V31225		
V31230		
V31267		
V31289		
V31295		
V31815		
V31927		
V31986		
